# Overexpression of decorin promoted angiogenesis in diabetic cardiomyopathy via IGF1R-AKT-VEGF signaling

**DOI:** 10.1038/srep44473

**Published:** 2017-03-14

**Authors:** Jinsheng Lai, Fuqiong Chen, Jing Chen, Guoran Ruan, Mengying He, Chen Chen, Jiarong Tang, Dao Wen Wang

**Affiliations:** 1 Division of Cardiology, Department of Internal Medicine and Gene Therapy Center, Tongji Hospital, Tongji Medical College, Huazhong University of Science and Technology, Wuhan, 430030, People’s Republic of China; 2 Department of Endocrinology, Tongji Hospital, Tongji Medical College, Huazhong University of Science and Technology, Wuhan 430030, P.R. China

## Abstract

Microcirculatory dysfunction is believed to play an important role in diabetic cardiomyopathy. The small leucine-rich proteoglycan decorin is generally considered a pro-angiogenic factor. Here, we investigate whether overexpression of decorin ameliorates diabetic cardiomyopathy and its effects on angiogenesis *in vivo* and *in vitro*. Diabetes was induced through intraperitoneal injection with streptozotocin combined with a high-fat diet, and decorin was overexpressed via recombinant adeno-associated virus in Wistar rats. Six months later, cardiac function was determined using an echocardiography and cardiac catheter system. The results showed that cardiac function was decreased in diabetic rats and restored by overexpression of decorin. In addition, overexpression of decorin upregulated the expression of VEGF and attenuated the reduction in the cardiac capillary density. In the *in vitro* study, high glucose induced apoptosis and inhibited the capabilities of tube formation, migration and proliferation, which were all ameliorated by decorin overexpression. Meanwhile, decorin overexpression increased the expression of VEGF and IGF1R, as well as the phosphorylation level of AKT and AP-1. Nonetheless, all of these effects were abolished by pretreatment with the IGF1R antibody or AKT inhibitor. In conclusion, overexpression of decorin ameliorated diabetic cardiomyopathy and promoted angiogenesis through the IGF1R-AKT-VEGF signaling pathway *in vivo* and *in vitro*.

Diabetes mellitus is a chronic disease with increasing worldwide prevalence and incidence, particularly in developing countries^1^. Diabetes mellitus has been reported to be an independent factor for cardiovascular diseases^2^, which are the leading cause of morbidity and mortality among diabetic patients^3^. One of these diseases is diabetic cardiomyopathy (DCM), which is defined as a disease secondary to diabetes mellitus in the absence of hypertension and coronary artery disease^4^.

DCM is characterized by myocardial cell apoptosis, hypertrophy, and interstitial fibrosis, as well as microvascular disorders. In addition, the microvascular disorders lead to myocardial ischemia disease, which finally develops into heart failure and causes death^5^. Studies carried out by Yoon *et al*. reported that the decreased capillary density and impaired myocardial perfusion in streptozotocin-induced diabetic rats could be reversed by replenishment of local vascular endothelial growth factor (VEGF)^6^. The expression of VEGF was regulated by several nuclear factors, such as HIF-1α^7^. Recently, Yu *et al*. reported that the expression of VEGF was regulated by AP-1, which was activated by the AKT/mTOR pathway^8^.

Moreover, the expression of VEGF has been reported to be able to be upregulated by decorin^9^. Decorin belongs to the family of small leucine-rich proteoglycans, which are characterized by a core protein with leucine-rich repeat motifs flanked by cysteine clusters and one chondroitin/dermatan-sulphate side chain^10^. Decorin is mainly expressed in mesenchymal cells such as fibroblasts^11^. Our previous studies revealed that overexpression of decorin inhibited cardiac fibrosis and hypertrophy via transforming growth factor-beta/Smad signaling^12^. Consistently, the evidence suggests that decorin might be potentially involved in reverse cardiac remodeling by directly inhibiting the TGF-β pathway and its pro-fibrotic effects on the failing human heart^13^. In addition, it has been reported that decorin promotes angiogenesis^9^. Consistently, Schonherr *et al*. also revealed that decorin promotes tube formation in endothelial cells^14^, which is the main process in angiogenesis. Decorin has also been reported to be important for angiogenesis in the cornea, and decorin deficiency leads to impaired angiogenesis in the injured mouse cornea^15^.

However, the effects of decorin on angiogenesis are controversial, since other studies have suggested that decorin acts as an antiangiogenic factor and suppresses VEGF activity^16^. Moreover, in a tumor, decorin-expressing cells produced VEGF at markedly reduced rates compared with their wild-type counterparts^17^. The difference in angiogenesis might reflect a context-dependent response to decorin.

Thus, it would be interesting to explore whether decorin can promote angiogenesis in DCM. To verify our hypothesis, we constructed a DCM model in rats by streptozotocin injection companied with a high-fat diet. Then, decorin was overexpressed through a recombined adeno-associated virus (rAAV) system, and the underlying mechanism was explored using the HUVEC cell line. In the current study, we showed that decorin played a protective role in DCM via promoting angiogenesis for the first time, which could be a new therapeutic strategy for patients suffering from DCM.

## Results

### Overexpression of decorin attenuated DCM

**Figure 1:**
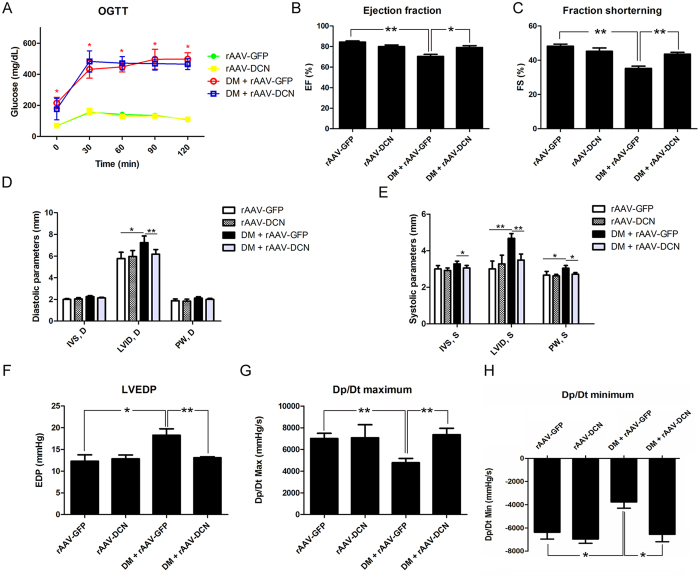
Overexpression of decorin improved cardiac function in diabetic cardiomyopathy. (**A**) The oral glucose tolerance test (OGTT). (**B**) The left ventricular ejection fraction (EF) evaluated by echocardiography. (**C**) The fraction shortening (FS) evaluated by echocardiography. (**D**,**E**) The left ventricular wall thickness (including the interventricular septum (IVS) and the posterior wall (PW)) and the internal dimension of the left ventricle (LVID). (**F**–**H**) The hemodynamic function was evaluated by the cardiac catheter system, including the left ventricular end-diastolic pressure (LVEDP), as well as the Dp/dt maximum and minimum. All data are presented as the mean ± SEM. *p < 0.05; **p < 0.01.

Forty rats were randomly divided into four groups. Streptozotocin and high-fat diet were used to induce diabetes, which finally developed into DCM. Decorin was delivered through the recombined adeno-associated virus system. As shown in Fig. 1A, the blood glucose of animals that received streptozotocin treatment was higher than that of the control group, indicating that diabetes mellitus had developed. Subsequently, these animals were subjected to echocardiograph and cardiac catheter examination. The results showed that compared to control rats, the ejection fraction (EF) that was decreased in diabetic rats was elevated to normal levels by overexpression of decorin (Fig. 1B). A similar effect was observed in the fraction shortening (FS) measurement (Fig. 1C). The internal dimension of the left ventricle (LVID) was increased in the diabetes group and ameliorated by overexpression of decorin (Fig. 1D and E). Additionally, the results from the cardiac catheter indicated that overexpression of decorin improved the left ventricular end-diastolic pressure (LVEDP), dp/dt maximum and dp/dt minimum (Fig. 1F–H), which were impaired in diabetic rats. Furthermore, cardiac fibrosis was assessed by Masson staining, and the results indicated that there was severe fibrosis in the diabetic hearts, which was attenuated by overexpression of decorin (Fig. S1). Taken together, the diabetic group had developed DCM, while overexpression of decorin attenuated the cardiac dysfunction and myocardial fibrosis.

### Overexpression of decorin upregulated the expression of VEGF and promoted angiogenesis *in vivo*

**Figure 2:**
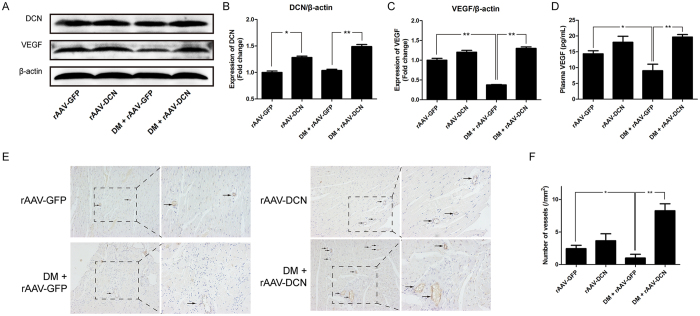
Overexpression of decorin increased angiogenesis in the diabetic hearts. (**A**–**C**) The expression of decorin and VEGF. (**D**) The VEGF concentration in the plasma. (**E**) The capillary density using immunochemistry with a CD31 antibody. The photographs were taken at a magnification of 100× and zoomed at a magnification of 200×. The arrows show the capillary stained with the CD31 antibody. (**F**) The number of vessels counted from the immunochemistry staining. All data are presented as the mean ± SEM. *p < 0.05; **p < 0.01.

As shown in Fig. 2A and B, the expression of decorin in the heart was upregulated after treatment with rAAV-DCN both in the control and diabetic rats. However, the expression of VEGF was decreased in the diabetic hearts and rescued by the overexpression of decorin (Fig. 2A and C). Consistently, compared to that of the control rats, the concentration of VEGF in the plasma of diabetic rats was decreased, which was then increased in the decorin-overexpressing rats (Fig. 2D). Immunohistochemical staining with CD31 was used to evaluate the capillary density. The results showed that the capillary density was reduced in diabetic hearts, whereas overexpression of decorin increased the capillary density in the diabetic hearts, as well as in the normal hearts (Fig. 2E and F).

### Overexpression of decorin promoted angiogenesis *in vitro*

**Figure 3:**
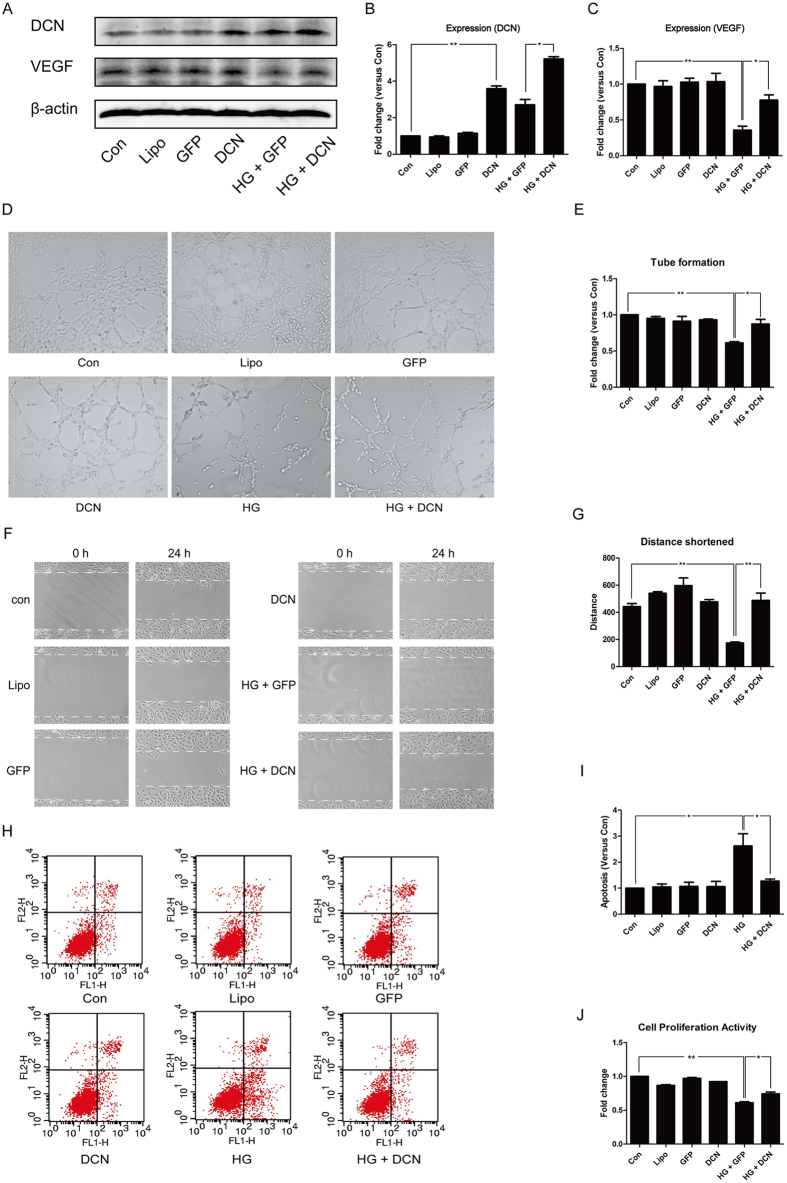
Overexpression of decorin ameliorated the angiogenesis impaired by high glucose (HG). (**A**–**C**) The expression of decorin and VEGF. (**D**,**E**) The tube formation test; the photographs were taken at a magnification of 100×. (**F**,**G**) The cell wound healing test; the photographs were taken in a magnification of 100×. (**H**,**I**) The apoptosis rate analyzed by an Annexin V–FITC kit through flow cytometry. (**J**) The proliferative ability assessed with a CCK8 assay. All data are presented as the mean ± SEM. *p < 0.05; **p < 0.01.

HUVECs treated with high glucose (HG) were investigated *in vitro*. The results showed that, compared to the control group, the expression of decorin increased significantly after transfection (Fig. 3A and B). The reduced expression of VEGF by HG treatment was ameliorated by overexpression of decorin (Fig. 3A and C).

Moreover, we evaluated the function of the endothelial cells, including the capability of tube formation, migration, and proliferation, as well as the apoptosis level. As shown in Fig. 3D and E, HG treatment decreased the tube formation capability, while overexpression of decorin attenuated this reduction. Interestingly, neutralizing VEGF with specific antibodies abolished the effects induced by decorin overexpression (Fig. S2).

Furthermore, a cell wound healing assay was employed in the assessment of migration. The results revealed that HG treatment inhibited endothelial cell migration, which was improved by overexpression of decorin (Fig. 3F and G). From the results shown in Fig. 3H and I, we noticed that overexpression of decorin decreased the apoptosis induced by HG. Meanwhile, a CCK8 assay analysis showed that HG impaired the proliferative capability of the cell, and overexpression of decorin attenuated it (Fig. 3J).

### Overexpression of decorin activated AKT and induced the expression of IGF1R

**Figure 4:**
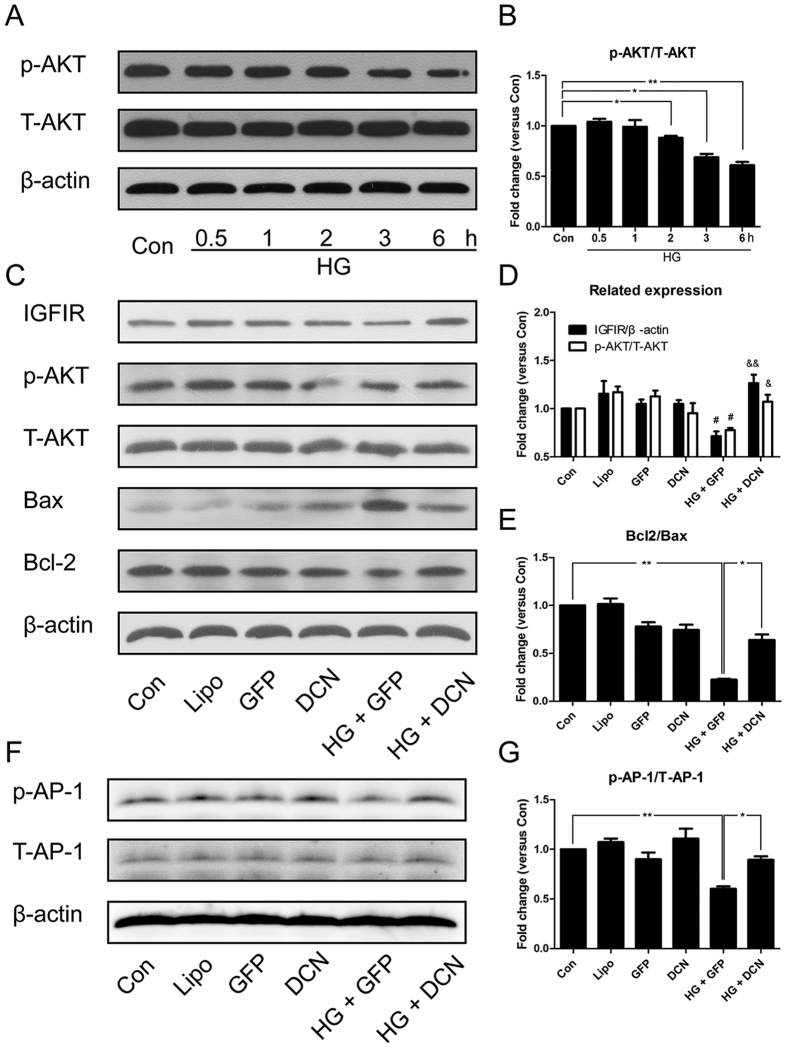
Overexpression of decorin activated the IGF1R-AKT-AP-1 pathway. (**A**,**B**) The phosphorylation of AKT was decreased by HG treatment in a time-dependent manner. (**C**–**E**) The expression of IGF1R, Bcl2, and Bax and the phosphorylation of AKT. (**F**,**G**) The phosphorylation of AP-1. All data are presented as the mean ± SEM. *p < 0.05; **p < 0.01. ^#^p < 0.05, compared to Con. ^&^p < 0.05, ^&&^p < 0.01, compared to HG + GFP.

As shown in Fig. 4A–D, HG reduced the phosphorylation of AKT in a time-dependent manner. In addition, this reduction was still observed 24 hours after treatment and could be reversed by overexpression of decorin. In addition, we analyzed the expression of the apoptosis-related proteins Bcl2 and Bax; the results indicated that HG increased the expression of Bax and downregulated the expression of Bcl2, which was attenuated by overexpression of DCN (Fig. 4B and D). In addition, the expression of IGF1R was analyzed; the results indicated that the expression of IGF1R was decreased by HG treatment, and overexpression of decorin restored IGF1R expression to normal levels (Fig. 4C and D). Moreover, the phosphorylation of AP-1 showed a similar tendency (Fig. 4D and E).

### The AKT inhibitor MK2206 abolished the effects induced by overexpression of decorin

**Figure 5:**
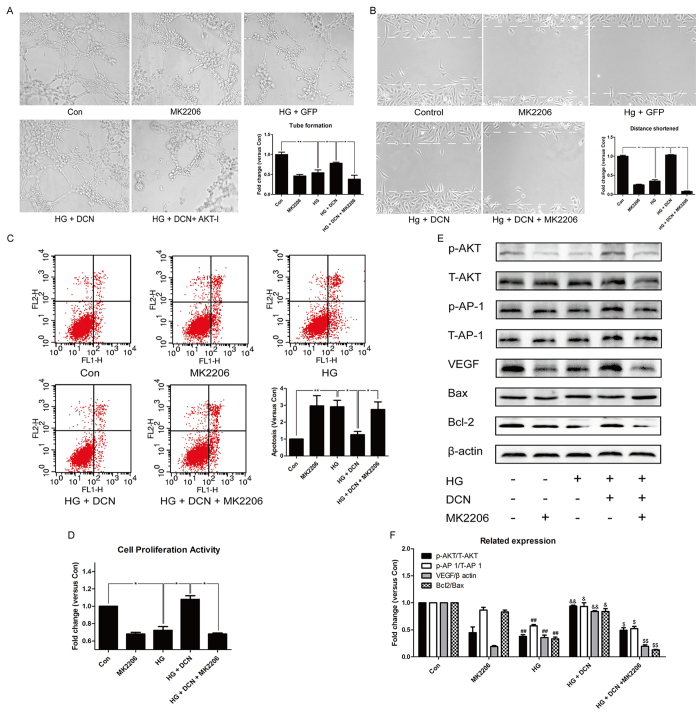
The AKT inhibitor MK2206 abolished the effects induced by overexpression of decorin. (**A**) The tube formation test; the photographs were taken at a magnification of 100×. (**B**) The cell wound healing test; the photographs were taken at a magnification of 100×. (**C**) The apoptosis assay. (**D**) CCK8 assessment. (**E**,**F**) The expression of VEGF, Bcl2, and Bax and the phosphorylation of AKT and AP 1. All data are presented as the mean ± SEM. *p < 0.05; **p < 0.01. ^#^p < 0.05, ^##^p < 0.01, compared to Con. ^&^p < 0.05, ^&&^p < 0.01, compared to HG + GFP (HG). $ p < 0.05, ^$$^p < 0.01, compared to HG + DCN.

To determine whether AKT participated in the VEGF regulation and angiogenesis induced by decorin, we used the AKT-specific inhibitor MK2206 to inhibit the activity of AKT. As shown in Fig. 5A, the tube formation ability of the cells was increased by overexpression of decorin, which was remarkably decreased after treatment with MK2206. Similar effects were observed in the cell wound healing assay (Fig. 5B) and apoptosis assessment (Fig. 5C) as well as the proliferative assay (Fig. 5D). The western blot results revealed that the activity of AKT was significantly reduced by MK2206, and a similar change occurred to its downstream effector AP-1 (Fig. 5E and F). From the ratio of Bcl2/Bax, inhibition of AKT obviously induced apoptosis in the group receiving MK2206 treatment (Fig. 5E and F). More importantly, the expression of VEGF was restored by overexpression of decorin, whereas the addition of MK2206 abolished these effects (Fig. 5E and F).

### IGF1R blockade disrupted the effects induced by overexpression of decorin

**Figure 6:**
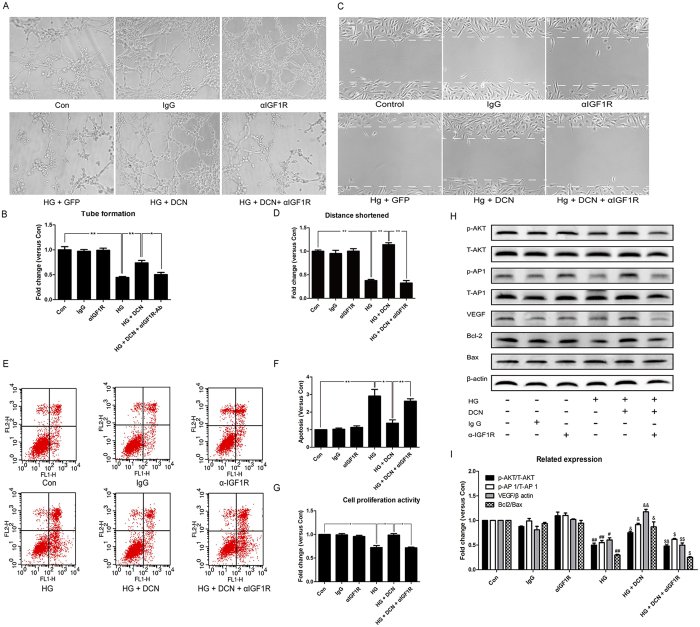
The IGF1R antibody (αIGF1R) blocked the effects induced by overexpression of decorin. (**A**,**B**) The tube formation test; the photographs were taken at a magnification of 100×. (**C**,**D**) The cell wound healing test; the photographs were taken at a magnification of 100×. (**E**,**F**) The apoptosis assay. (**G**) CCK8 assessment. (**H**,**I**) The expression of VEGF, Bcl2, and Bax and the phosphorylation of AKT and AP 1. All data are presented as the mean ± SEM. *p < 0.05; **p < 0.01. ^#^p < 0.05, ^##^p < 0.01, compared to Con. ^&^p < 0.05, ^&&^p < 0.01, compared to HG + GFP. ^$^p < 0.05, ^$$^p < 0.01, compared to HG + DCN.

To further investigate the role of IGF1R in decorin-driven angiogenesis, we adopted an IGF1R-specific antibody to block IGF1R. The results indicated that the capability of tube formation, migration and proliferation, as well as the apoptosis level of the cells, were ameliorated by the overexpression of decorin after HG treatment. However, these effects were blocked by the addition of IGF1R blockade using the IGF1R antibody (Fig. 6A–G). Moreover, as shown in Fig. 6H and I, blocking IGF1R with the antibody decreased the phosphorylation level of AKT and AP-1, as well as the expression of VEGF. In addition, apoptosis assessment by the ratio of Bcl2/Bax was also decreased by IGF1R blockade, which was then increased by decorin (Fig. 6H and I).

## Discussion

**Figure 7:**
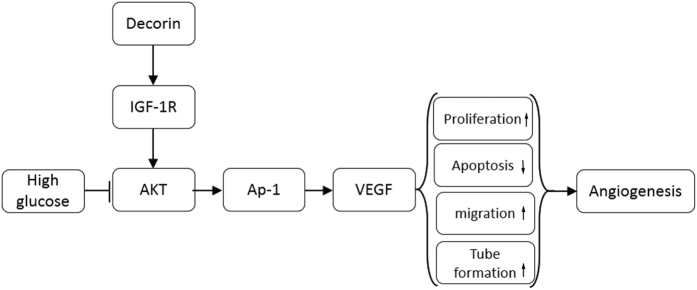
Schematic of mechanisms of decorin-mediated signaling in high glucose-impaired angiogenesis. In endothelial cells, decorin overexpression activates IGF1R and promotes the phosphorylation of AKT and AP-1, which is inhibited by high glucose or hyperglycemia. The activation of AP-1 will upregulate the expression of VEGF, which increases proliferation, decreases apoptosis, and promotes migration and tube formation of the cells. Finally, all of these processes contribute to the promotion of angiogenesis and ameliorate DCM.

In the present study, we used streptozotocin and high-fat diet to induce diabetes in rats. In addition, an echocardiograph and cardiac catheter system were used to assess the cardiac function *in vivo*. Capillary density and fibrosis were evaluated. The results indicated that the diabetic rats progressed to DCM, including impaired cardiac function, decreased capillary density and severe cardiac fibrosis, whereas overexpression of decorin attenuated these effects. Overexpression of decorin increased the migratory ability, proliferative activity, and the tube formation capability, which were damaged by high glucose treatment *in vitro*. In addition, high glucose induced cell apoptosis, which was prevented by decorin overexpression (Fig. 7).

Overexpression of decorin has been reported to mitigate cardiac remodeling and dysfunction in infarcted hearts^18^. We first hypothesized that decorin could attenuate cardiac remodeling in DCM via promoting angiogenesis. DCM is characterized by cardiac dysfunction, low capillary density and interstitial fibrosis^19^,^20^, which was further confirmed in our study. Moreover, we showed that overexpression of decorin increased the capillary density in the diabetic hearts and decreased fibrosis. This effect caused by decorin has also been observed in other models. In an oral cancer model, decorin was aberrantly expressed in the cancer cells, and knockdown of decorin reduced angiogenic mediators, including VEGF and MMP9, and decreased angiogenesis^21^. Furthermore, Chui *et al*. reported that in fetal growth restriction, a short interference RNA-induced reduction in decorin decreased proliferation and angiogenesis in endothelial cells^22^. In accordance with these findings, in mouse cerebral endothelial cells, ectopic decorin expression could increase angiogenesis through the EGFR/ERK and EGFR/AKT pathways^9^.

However, there have been some other studies that supported another possibility. Studies by Rajiv *et al*. suggested that rAAV5-mediated decorin expression downregulated expression of VEGF and MCP1 and decreased angiogenesis in the cornea^23^. In addition, decorin has been reported to be able to reduce the expression of VEGF at the mRNA and protein level and decrease angiogenesis in tumor cells^17^. Hence, we noticed that decorin played a different role on the effects of angiogenesis in different models. In the present study, we report for the first time that decorin has a pro-angiogenesis effect in DCM and clarified the mechanism of this effect.

Our results first showed that overexpression of decorin ameliorated the level of apoptosis and capabilities of tube formation, migration, and proliferation, which were damaged by high glucose treatment in endothelial cells. However, these effects could be disrupted when IGF1R was blocked. The data suggest that the pro-angiogenesis effect of decorin in DCM may be related to IGF1R. Consistent with our findings, Schonherr *et al*. reported that decorin could bind IGF1R and activate its tyrosine kinase activity in endothelial cells. In addition, the binding assay showed only a 10-fold lower affinity of decorin to the IGF1R than IGF-1^24^. Coincidentally, decorin has been reported to modulate endothelial cell behavior through activation of IGF1R^25^.

Once IGF1R has been activated, its tyrosine kinase activates the downstream effectors^26^,^27^. One of them is AKT, which is important for cell survival and proliferation^28^. Our results suggested that AKT was activated by IGF1R and responsible for VEGF expression. VEGF is believed to be the major pro-angiogenesis factor, and the expression of VEGF is regulated by several factors^7^. In the present study, decorin enhanced VEGF expression via AP-1, which was activated by AKT. Similarly, studies by Huang *et al*. reported that inhibition of AP-1 activation decreased the expression of VEGF^29^. In addition, the VEGF receptor activated by VEGF in turn increased AKT activation and promoted endothelial cell survival^30^.

However, it should be noted that this study still had some defects. First, we observed that decorin could promote angiogenesis, but we do not know which domain of decorin participated in this action, since there are four domains in the decorin core protein^31^. Second, decorin is known to be a ligand for several cell surface receptors including EGFR^32^, the hepatocyte growth factor (HGF) receptor Met^33^,^34^, IGF1R^24^,^27^,^35^ and VEGFR2^36^, but we only focused on the relationship between decorin and IGF1R. Therefore, more work should be carried out to study the relationship between decorin and the other receptors.

## Conclusions

In conclusion, the results conclusively identify decorin as a protective agent in DCM. Moreover, overexpression of decorin protected endothelial function from hyperglycemia and promoted angiogenesis through IGF1R/AKT/AP-1/VEGF signaling, suggesting that decorin could be a new therapeutic strategy for patients suffering from DCM.

## Methods

### Material and reagents

Glucose and MK2206 were bought from Sigma-Aldrich (Sigma-Aldrich, Missouri, USA). Antibodiesagainst CD31 and decorin were bought from Boster (Boster, Wuhan, China), while antibodies against VEGF, p-AKT, T-AKT, p-c-JUN, T-c-jun and β-actin were purchased from Santa Cruz biotechnology (Santa Cruz, Texas, USA). Two antibodies against IGF1R were used. One was used for western blot and the other for neutralizing IGF1R. The former was a rabbit polyclonal antibody purchased from Boster that reacts with rat and human, as well as mouse, IGF1R. The latter antibody, used for neutralizing IGF1R, was purchased from Abcam (Abcam, Cambridge, UK) and blocks IGF1 by binding to its receptor. It is a mouse monoclonal antibody against the IGF1 receptor and mainly reacts with human IGF1R. The ECM gel was purchased from BD Bioscience (BD Bioscience, CA, USA). All other reagents were purchased from commercial suppliers unless otherwise indicated.

### Experimental animals

Forty male Wistar rats were randomly divided into 4 groups, the control group (rAAV-green fluorescent protein (GFP)), the decorin overexpression group (rAAV-DCN), the diabetes mellitus group (DM + rAAV-GFP, which developed DCM), and the DM rats with overexpression of decorin group (DM + rAAV-DCN). All animals were raised in the animal center of Tongji Medical College, Huazhong University of Science and Technology. The study protocol was approved by the committee on the Ethics of Animal Experiments of the Animal Research Committee of Tongji Medical College. The procedures used in the animal treatments complied with the NIH Guide for the Care and Use of Laboratory Animals (1996). All efforts were made to minimize suffering. All rats were housed under standard laboratory conditions (12-hour light: 12-hour dark cycle, 22–25 °C room temperature, and 45–55% relative humidity). Diabetes was induced through intraperitoneal injection with streptozotocin (Sigma-Aldrich, Missouri, USA) at a dose of 40 mg/kg combined with a high-fat diet (HFK Bioscience, Beijing, China) as described previously^37^. Decorin was overexpressed by the recombinant adeno-associated virus serotype 9 (rAAV-9) system, which transduces heart cells with high efficiency^38^.

### Oral glucose tolerance test

The oral glucose tolerance test (OGTT) was conducted to diagnose diabetes. After fasting for 12 hours overnight, the blood samples were collected from the tail vein^39^. Following blood collection, the rats were fed with 50% glucose through a gastric tube at a dose of 2 g/kg body weight. After 30 min, 60 min, 90 min and 120 min, the blood samples were collected. Then, the samples were isolated after centrifuging at 1600 g for 10 min. The blood glucose level was determined using an AEROSET Clinical Chemistry System (Abbott Laboratories, Abbott Park, Illinois).

### Echocardiography

The cardiac function of the animals was evaluated through a VIVO 2100 echocardiography machine (VisualSonics, Toronto, Canada). Before the detection, the chest hair of the rats was removed with a pet razor. Then, the animals were anesthetized with intraperitoneal injections of a xylazine (5 mg/kg) and ketamine (100 mg/kg) mixture and tied to expose the chest area. A 30-MHz probe was used to carry out the exam. The ejection fraction (EF), fraction shortening (FS), thickness of the left ventricular wall, and the internal dimension were determined and analyzed.

### Hemodynamic assessment

The hemodynamics of the heart were evaluated by the Millar pressure volume system (MPVS) (ADinstruments, New South Wales, Australia), which has been described previously^40^. Briefly, after the animals were anesthetized and fixed, the right carotid artery was separated. The distal artery was obligated, and the proximal was blocked with an artery clamp. A suitable incision was made with microscissors, and the clamp was released after the duct was inserted into the artery. The duct was then advanced into the left ventricle. The data were recorded for at least 20 min after the wave stabilized. The Dp/dt maximum and minimum and the end-diastolic pressure were analyzed using the MPVS system.

### Immunohistochemical staining

After the animals were killed, the hearts were separated, fixed with 4% neutral formalin, dehydrated and prepared in paraffin sections. Sections were incubated with the appropriate CD31 antibody, and a 0.3% hydrogen peroxide solution was employed to block the endogenous peroxidase activity. Following application of the appropriate biotinylated secondary antibody, sections were developed with the DAB substrates. The photographs were captured with a Nikon inverted microscope (Nikon, Tokyo, Japan), and the number of vessels was measured with Image-Pro Plus 6.

### Masson staining

Masson staining was used to evaluate fibrosis in the hearts of the rats. The hearts were fixed with neutral formalin, sectioned, dehydrated, prepared in paraffin sections, and stained using a Masson staining kit (Jiancheng Bioengineering Institute, Nanjing, China). The Masson staining were performed according to manufacturer’s protocol. Collagen fibers were in blue, and the cells were in red. The photographs were captured with a Nikon inverted microscope (Nikon, Tokyo, Japan).

### Cell culture

The human umbilical vein endothelial cells (HUVECs) obtained from the American Type Culture Collection were cultured in RPIM 1640 with 10% FBS. These cells were digested with 0.04 g/ml 0.25% trypsin-ethylene diamine tetraacetic acid (EDTA) and transferred to 6-well plates. For each well, 1 × 10^6^ cells were planted. A Lipo 2000 transfection kit was used to overexpress decorin in these cells. Twenty-four hours after transfection, glucose or mannitol was added to the cells at a final concentration of 30 mM.

### Tube formation assay

The tube formation assay was used to assess *in vitro* angiogenesis. In brief, 100 μL BD Matrigel was planted into precooled 96-well plates on ice and incubated for 30 min at 37 °C. Then, the HUVECs were digested and transferred to the wells. For each well, 3 × 10^4^ cells were planted in 100 μL medium. Then, the cells were incubated at 37 °C for 4 hours, and the endothelial tubes were observed using a light microscope. Photographs were captured with a Nikon inverted microscope (Nikon, Tokyo, Japan), and the number of tubes was counted and analyzed.

### CCK8 assay

A cell counting kit 8 (CCK8) assay was carried out using a CCK8 assay kit (Boster, China) to evaluate the proliferative activity following the manufacturer’s instructions. The cells were planted in 96-well plates and incubated in 100 μL fresh medium without FBS. Then, 10 μL CCK8 was added into the wells; at the same time, a blank well contained no cells but had the medium and CCK8. The plates were incubated at 37 °C for 2 hours before measuring. The absorbance in 450 nm was determined. The proliferative activity was calculated with the formula below:


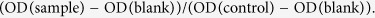


### Cell wound healing test

The cell wound healing test, also known as a scratch assay, was employed to assess the migratory ability of the cells. The HUVECs were planted into 6-well plates. After the cells covered 80% of the bottom, the plasmid carrying the decorin gene was transfected into the cells. Twenty-four hours later, a 100-μl sterilized tip was used to create a scratch in the cells. For each well, 5 scratches were made, and the tip was perpendicular to the bottom of the plate every time to make sure that all scratches were of the same width. The cells were washed with PBS 3 times and incubated in incomplete medium at 37 °C for 24 hours. Photographs were captured with an Olympus inverted microscope. The width of every scratch was measured and analyzed with Image Pro-Plus software.

### Annexin V-Fluorescein Isothiocyanate Apoptosis Assay

Apoptosis of the cells was assessed through flow cytometry using the Annexin V- fluorescein isothiocyanate (FITC) and propidium iodide kit (Keygen, Nanjing, China). After treatment, cells were digested with trypsin and washed with PBS 3 times. In addition, cells were resuspended with 500 μL binding buffer. According to the manufacturer’s protocol, 5 μL Annexin V-FITC and 5 μL propidium iodide were added and incubated for half of an hour. Cells were then analyzed with a FACStar-Plus flow cytometer (Becton Dickinson, CA, USA).

### Western blotting

Cells were lysed with RIPA lysate (Beyotime, Shanghai, China) and centrifuged at 12000 g for 20 min. The protein concentration was measured with a BCA protein assay. Then, 50 μg protein for each well was loaded into the 10% SDS-PAGE gel, followed by transfer to a polyvinylidene difluoride membrane (Bio-Rad, California, USA). The membrane was blocked with 5% BSA and incubated with 1:1000 primary antibody. After incubation for at least 16 hours, the membrane was washed with Tris-NaCl solution 6 times and incubated with 1:10000 horseradish peroxidase-conjugated secondary antibody for two hours. The membrane was washed again with the Tris-NaCl solution and developed with ECL (Advansta, California, USA). The photograph was captured with the Luminescent Imaging System (Tanon, Shanghai, China).

### Statistical analysis

All of the experiments were performed at least three times independently, and the data were presented as the mean ± standard error of measurement (SEM). A one-way analysis of variance (ANOVA) was used to assess the data. When p < 0.05, it was considered statistically significant.

## Additional Information

**How to cite this article:** Lai, J. *et al*. Overexpression of decorin promoted angiogenesis in diabetic cardiomyopathy via IGF1R-AKT-VEGF signaling. *Sci. Rep.* **7**, 44473; doi: 10.1038/srep44473 (2017).

**Publisher's note:** Springer Nature remains neutral with regard to jurisdictional claims in published maps and institutional affiliations.

## Supplementary Material

Supplementary Information
