# Supplementary material for: Overexpression of decorin promoted angiogenesis in diabetic cardiomyopathy via IGF1R-AKT-VEGF signaling
Source: Sci Rep. 2017 Mar 14;7:44473. doi: 10.1038/srep44473 (PMC5349602; doi:10.1038/srep44473)
Supplement: Supplementary Information [file srep44473-s1.doc]

**Overexpression of decorin promoted angiogenesis in diabetic cardiomyopathy via IGF1R-AKT-VEGF signaling**

Jinsheng Lai^1#^, Fuqiong Chen^2#^, Jing Chen^1^, Guoran Ruan^1^, Mengying He^1^, Chen Chen^1^, Jiarong Tang^1^*, Dao Wen Wang^1^

^1^Division of Cardiology, Department of Internal Medicine and Gene Therapy Center, Tongji Hospital, Tongji Medical College, Huazhong University of Science and Technology, Wuhan, 430030, People’s Republic of China.

^2^Department of Endocrinology, Tongji Hospital, Tongji Medical College, Huazhong University of Science and Technology, Wuhan 430030. PR China.

^#^ These authors contributed equally to this work.

* To whom correspondence should be addressed:

Jiarong Tang, M.D.

Division of Cardiology, Department of Internal Medicine and Gene Therapy Center,

Tongji Hospital, Tongji Medical College

Huazhong University of Science & Technology

1095# Jiefang Ave., Wuhan 430030 PRC

Tel. and Fax: 86-27-8366-3280

Emial: [jrtang@tjh.tjmu.edu.cn](mailto:jrtang@tjh.tjmu.edu.cn)

**Figure S1**

**
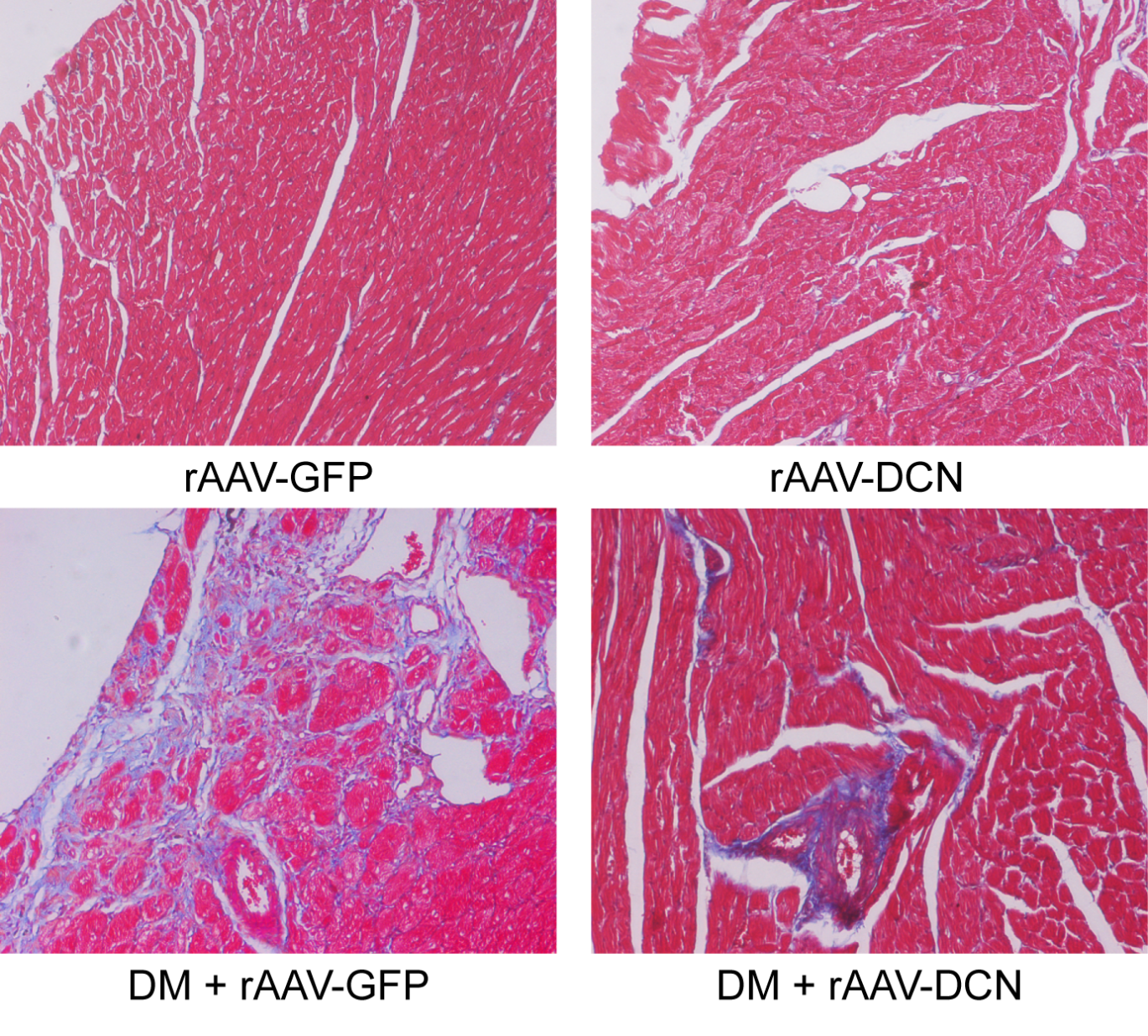
**

**Figure S1. The fibrosis level of the hearts.** The Masson staining was used to evaluate the fibrosis level in the hearts of the rats. Compared to the control rats (rAAV-GFP) group, the diabetic rats developed severe cardiac fibrosis, which was attenuated by overexpression of decorin. DM: diabetes mellitus, DCN: decorin. The photographs were taken in a magnification of 100×.

**Figure S2**


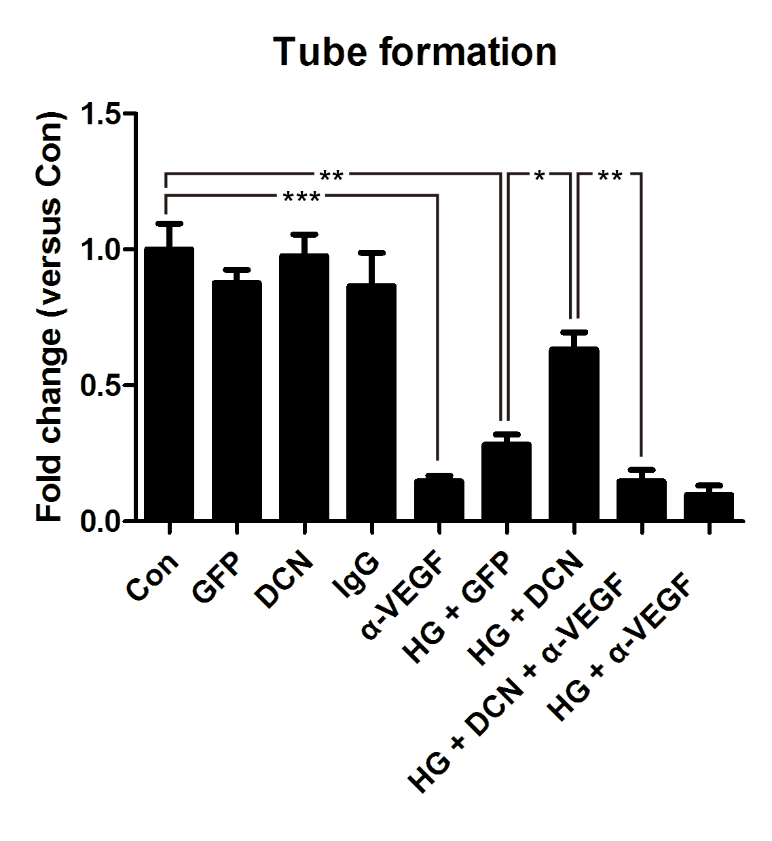


A

B


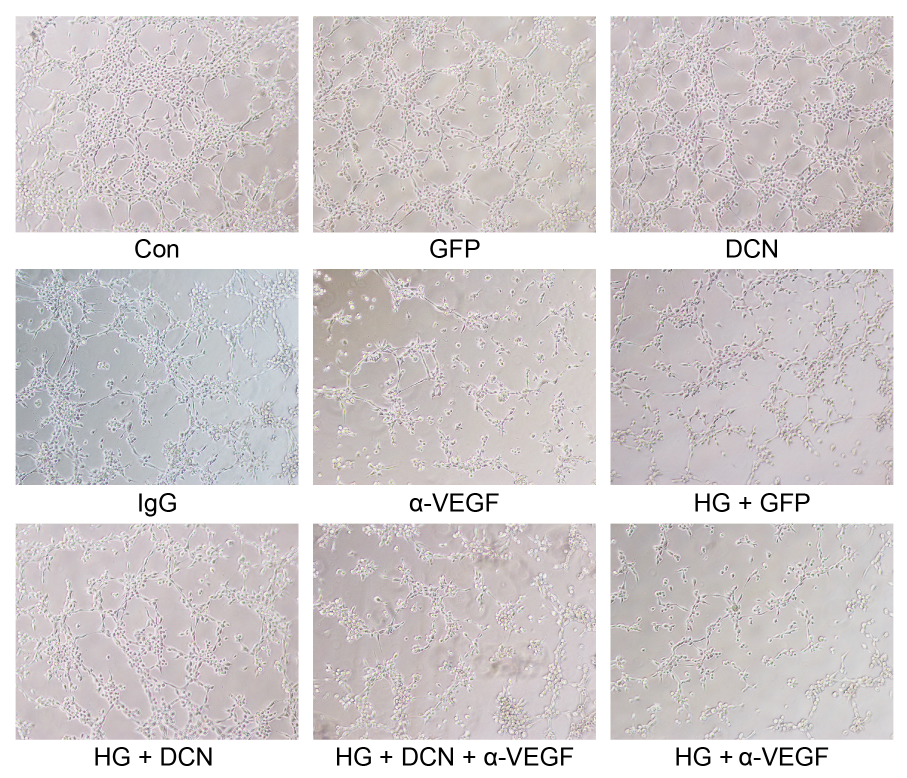


**Figure S2. VEGF antibody (α-VEGF) blocked the effects induced by overexpression of decorin.** (A, B) The tube formation test, the photographs were taken in a magnification of 100×. * p < 0.05.
